# Phase-dependent loading characteristics of the supporting-side knee joint during the flying kick with a 180-degree inward turn in competitive Tai Chi

**DOI:** 10.3389/fbioe.2026.1959458

**Published:** 2026-10-07

**Authors:** Pan Deng, Mengyao Jia, Yong Ma

**Affiliations:** 1 Key Laboratory of Sports Engineering of General Administration of Sport of China, Wuhan Sports University, Wuhan, China; 2 Research Center of Sports Equipment Engineering Technology of Hubei Province, Wuhan Sports University, Wuhan, China; 3 School of Intelligent Sports Engineering, Wuhan Sports University, Wuhan, China; 4 Research Center for High-Quality Development of Characteristic Competitive Sports, Wuhan Sports University, Wuhan, China

**Keywords:** competitive Tai Chi, finite element analysis, knee joint, landing biomechanics, phase-dependent characteristics

## Abstract

**Introduction:**

Competitive Tai Chi has increasingly incorporated high-difficulty aerial and single-leg landing techniques. The flying kick with a 180° inward turn (322C) is a demanding C-level movement that requires rapid take-off, aerial hand-foot slap, body rotation, and stable single-leg landing. However, the phase-dependent loading characteristics of the supporting-side knee joint during this movement remain unclear.

**Methods:**

Ten male competitive Tai Chi athletes performed the 322C while three-dimensional kinematics and ground reaction forces (GRF) were recorded. The movement was divided into four phases: take-off propulsion (P1), hand-foot slap (P2), flight end (P3), and landing cushioning (P4). Joint angles, moments, reaction forces, and vertical GRF (VGRF) were calculated using inverse dynamics. A subject-specific finite element (FE) model of the supporting-side knee was reconstructed from CT and MRI, and the peak landing loads were applied to evaluate internal tissue stresses.

**Results:**

P4 had the longest duration (1.639 s). VGRF reached 5.18 ± 1.84 body weight (BW), with a peak knee extension moment of 5.01 ± 1.64 N.m-kg^−1^ and a peak compressive joint reaction force of 22.58 BW. FE analysis showed non-uniform stress distributions, with the highest von Mises stress observed in the anterior cruciate ligament (ACL; 54.24 MPa), posterior cruciate ligament (41.79 MPa), medial meniscus (23.53 MPa), and lateral femoral cartilage (9.62 MPa).

**Discussion:**

The 322C imposes different mechanical demands on the supporting-side knee across phases. P1 is mainly characterized by rapid knee extension and load transfer, whereas P4 involves high impact, axial compression, shear, and rotational stabilization. The stress concentrations observed in the ACL and anterior regions of the menisci indicate areas of relatively high local loading during the movement. These findings provide biomechanical information for understanding knee loading during the 322C and may help inform technique optimization and injury-prevention strategies in competitive Tai Chi.

## 1 Introduction

According to current Wushu routine competition rules, competitive Tai Chi has incorporated a substantial number of high-difficulty jumping, aerial kicking, and rotating maneuvers. Among these, the 322C is classified as a C-level difficulty element and is regarded as a key indicator of an athlete’s technical proficiency and special motor ability (Zhou and Liu, 2010; Zhu, 2021). This movement requires the athlete to complete take-off, aerial hand-foot slap contact, body rotation, and stable single-leg landing within a very short time window (Fang, 2016). Unlike conventional double-leg landing tasks, the 322C involves landing immediately after aerial rotation, which means the supporting side must absorb a large impact load while simultaneously performing cushioning, rotation braking, postural stabilization, and center-of-mass adjustment. In competitive Tai Chi routines, this movement often serves as a transitional element linking other techniques, and thus its landing quality directly affects the overall stability, continuity, and expressive quality of the entire routine. However, single-leg landing combined with high impact and rotational inertia may substantially increase the mechanical burden on the supporting-side knee joint and potentially elevate injury risk.

The knee joint is a key load-bearing structure within the lower-limb kinetic chain. During jump landing, it not only performs sagittal-plane flexion-extension cushioning but also contributes to frontal-plane stability and transverse-plane rotational control (Luan et al., 2025). Previous biomechanical studies have demonstrated that knee flexion angle, joint moments, trunk posture, center-of-mass control, and lower-limb muscle coordination patterns during landing all affect impact absorption and load transfer, thereby influencing internal joint loading (Yuan, 2010; Zeng, 2016). Insufficient knee flexion, poor trunk control, or impaired neuromuscular coordination may increase abnormal stress on structures such as the ACL, menisci, and articular cartilage (Molloy et al., 2025). In the field of competitive Tai Chi, existing biomechanical studies have mainly focused on the kinematic and kinetic characteristics of difficult movements such as the aerial lotus kick and the tornado kick. For example, studies on the 360° aerial lotus kick followed by knee-lift balance have reported that sufficient hip, knee, and ankle flexion during take-off, combined with coordinated arm swing, contributes to improved aerial performance, while the lower-limb cushioning strategy during landing is essential for movement stability (Fu, 2019). Similarly, research on the 360° tornado kick has shown that swinging-leg velocity, trunk-upper limb coordination, and the control ability of the supporting side during landing all influence rotation efficiency and landing stability (Chao, 2011). However, compared with these movements, the 322C places greater emphasis on the transition between aerial kicking and single-leg landing. Its movement pattern and loading characteristics may therefore differ substantially from other Tai Chi difficulty movements, yet the phase-dependent knee loading during this specific maneuver remains poorly understood.

In addition to jumping techniques, previous studies have investigated lower-limb biomechanics during Tai Chi stepping and pushing-hand movements. Kinematic, kinetic, and surface electromyographic studies have shown that Tai Chi gait generally involves a longer movement cycle, greater joint range of motion, and more pronounced muscle activation than normal walking (Tseng et al., 2007; Wu et al., 2004). Studies focusing on the knee joint have further revealed that different postures and stances significantly affect knee loading. For instance, low-posture Tai Chi movements are usually associated with larger knee extension moments, whereas higher postures may reduce some unfavorable frontal-plane loads. Conversely, incorrect execution of techniques such as Parting the Wild Horse’s Mane may increase knee abduction and external rotation moments, potentially aggravating wear of internal structures (Wen et al., 2018; Wang et al., 2022). However, it should be noted that these studies are still largely based on external measurements, such as kinematics, kinetics, and surface electromyography, which cannot directly reveal stress, strain, or contact distribution within knee tissues during high-impact landing. For a complex movement such as the 322C, which involves single-leg landing, large impact forces, and rotational control, external experimental variables alone are insufficient to fully explain the internal load formation mechanism of the supporting-side knee joint. However, experimental biomechanical measurements primarily provide information on external kinetics and joint motion and cannot directly quantify the internal stress distribution within individual knee tissues. Therefore, finite element analysis provides a complementary approach for further investigating the internal mechanical responses of the knee joint during high-load movements.

Finite element (FE) analysis provides an effective tool for investigating internal joint mechanical responses and has been widely used in sports injury and orthopedic biomechanics. By integrating medical imaging data, material properties, and boundary/loading conditions, FE models can quantitatively evaluate stress and strain distributions in bones, cartilage, menisci, and ligaments under complex loading conditions. In recent years, FE methods have been applied to Tai Chi research, primarily focusing on typical stepping movements such as Brush Knee and Twist Step. These studies generally suggest that slow Tai Chi stepping produces relatively low joint contact stress compared with walking or jogging (Chang et al., 2021; Duan et al., 2019). However, most existing simulation studies have focused on slow and controllable stepping movements; the internal knee joint responses during high-difficulty jumping movements in competitive Tai Chi, particularly during landing, remain insufficiently investigated.

Therefore, the present study combined experimental biomechanical testing and FE simulation to systematically investigate the load characteristics of the supporting-side knee joint during the 322C in competitive Tai Chi. We collected kinematic and kinetic data across the entire movement cycle and reconstructed a subject-specific three-dimensional FE knee model from CT and MRI images. Experimentally derived loading conditions at the peak landing-cushioning moment were applied to quantify stress distribution in the femur, tibia, menisci, articular cartilage, and knee ligaments. By integrating external biomechanical variables with internal tissue-level mechanical responses, this study aimed to clarify the phase-dependent loading characteristics of the supporting-side knee, identify high-risk structures during landing, and provide theoretical guidance for technique optimization, specialized training, and knee injury prevention in competitive Tai Chi athletes.

## 2 Materials and methods

### 2.1 Study participants

Ten male competitive Tai Chi athletes were recruited from the Hubei provincial Wushu routine Tai Chi team. All athletes were at least national first-class level and had experience in provincial or higher-level competitions.

The inclusion criteria were as follows: 1) specialized training in competitive Tai Chi; 2) ability to complete the 322C according to competition standards; 3) no lower-limb sports injury within the previous 6 months; 4) no history of lower-limb surgery; and 5) no acute musculoskeletal injury or neurological disorder affecting movement performance. All participants were right-leg dominant and used the right leg as the supporting side during the movement; therefore, the right side was defined as the supporting side in this study.

The participants’ age, height, body mass, and training experience were as follows: age, (19.00 ± 0.81) years; height, (177.50 ± 3.75) cm; body mass, (66.30 ± 2.45) kg; and training experience, (9.71 ± 2.43) years. All participants provided written informed consent before testing. The study protocol was approved by the Medical Ethics Committee of Wuhan Sports University (No. 2023069).

### 2.2 Experimental setup and data collection

Before data collection, participants wore tight sports shorts and completed a standardized warm-up consisting of 5 min of treadmill running at 6 km/h with 0% incline, followed by 5 min of stretching. Each athlete then practiced the 322C once to become familiar with the experimental environment and the location of the force plate. After a 2-min seated rest, reflective markers were attached.

A total of 70 reflective markers with a diameter of 14 mm were placed on each participant, including 48 anatomical landmark markers and 22 auxiliary tracking markers. The marker set was based on the Vicon technical manual and was optimized according to the technical characteristics of the 322C.

Three-dimensional kinematic data were collected using a Vicon motion capture system (nine T40 infrared cameras at a sampling frequency of 250 Hz). GRF data were collected synchronously using a Kistler three-dimensional force plate model (9260AA6, Kistler, Switzerland at a sampling frequency of 1,500 Hz). The motion capture and force plate systems were temporally synchronized.

During formal testing, participants stood in the designated force plate area and maintained a standardized preparatory posture for 3–5 s. They then performed the 322C according to a unified verbal command. At least three successful trials were collected for each participant. A successful trial met the following criteria: 1) the movement was judged by qualified Wushu judges to meet competition standards; 2) the hand-foot slap contact moment was confirmed using synchronized video and marker trajectories; 3) the supporting foot landed completely on a single force plate; and 4) marker trajectories were complete and continuous. Failed trials were recorded and excluded from analysis.

### 2.3 Movement phase definition

According to the technical structure of the 322C movement, the complete movement cycle was divided into five key events and four phases (Figure 1).

E1: maximum crouch movement (lowest center of mass before take-off)
E2: take-off movement (GRF <10 N)
E3: Clap moment (hand contacted the dorsum of the kicking foot)
E4: landing moment (VGRF >10 N)
E5: maximum cushioning moment (lowest center of mass after landing)

**FIGURE 1:**
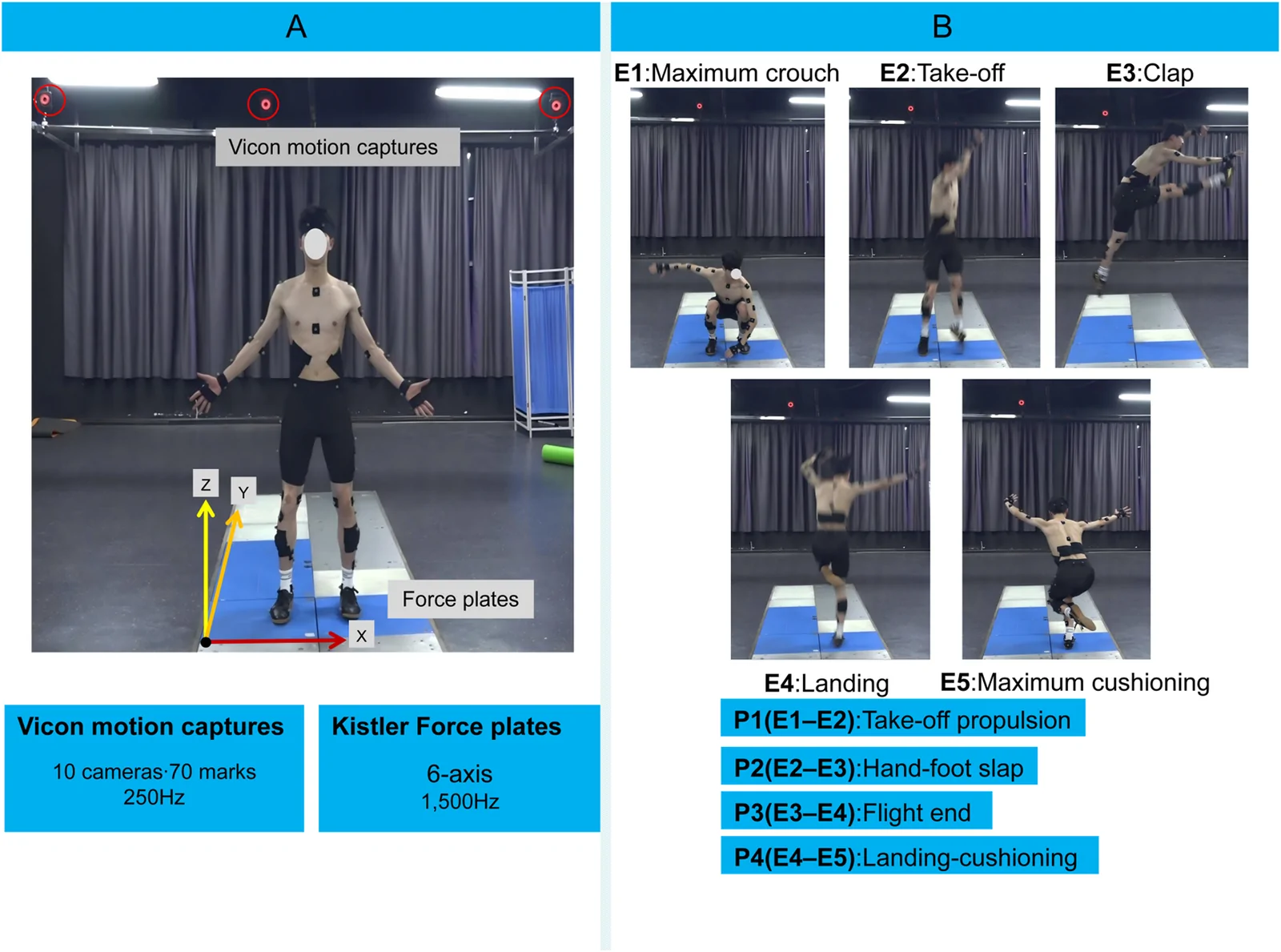
Experimental setup and phase delineation. **(A)** Laboratory environment **(B)** Movement phase definition.

Phases were defined as: P1 (E1–E2, take-off propulsion), P2 (E2–E3, hand-foot slap), P3 (E3–E4, flight end), and P4 (E4–E5, landing-cushioning).

### 2.4 Indicators

Based on the characteristics of the 322C and previous biomechanical studies (Fang, 2016) on difficult movements in competitive Tai Chi, the following indicators were selected. Kinematic indicators included movement duration, center-of-mass height, and joint angle (deg). Kinetic indicators included joint moment (N·m·kg^−1^), joint reaction force, normalized to body weight (BW), and VGRF (BW).

The joint coordinate system used for the calculations is shown in Figure 2. For knee joint angles, positive and negative values in the X-, Y-, and Z-directions represented extension and flexion, adduction and abduction, and internal and external rotation, respectively. For knee joint moments, positive and negative values in the X-, Y-, and Z-directions represented extension and flexion moments, adduction and abduction moments, and internal and external rotation moments, respectively. For knee joint reaction forces, positive and negative values in the X-direction represented anterior and posterior forces, respectively; those in the Y-direction represented medial and lateral forces, respectively; and those in the Z-direction represented tension and compression, respectively. For knee joint stress, the stress distribution (MPa) in the knee cartilage and ligaments was calculated using Abaqus.

**FIGURE 2:**
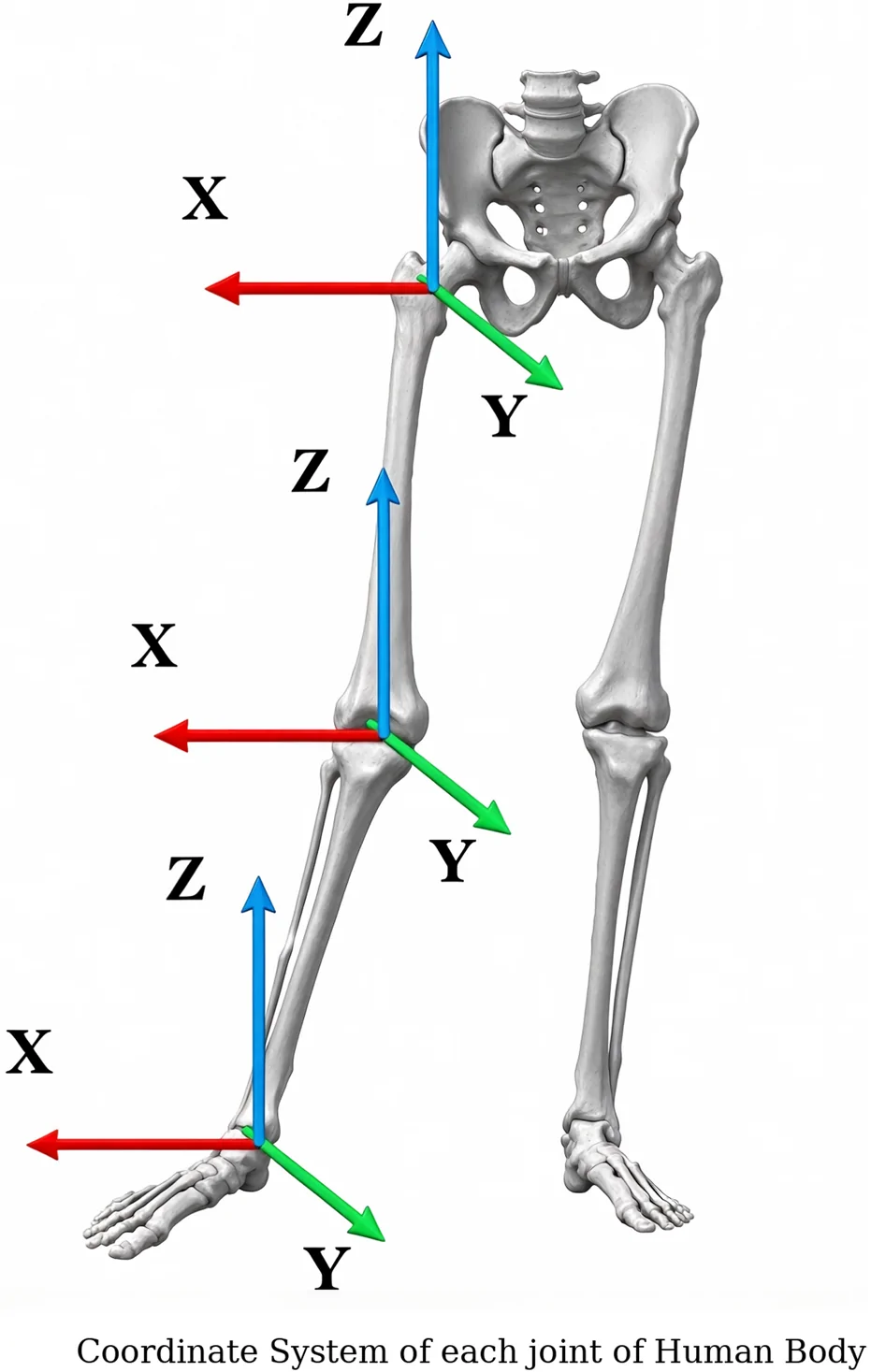
Schematic diagram of the joints and coordinate systems.

### 2.5 Experimental data processing

Kinematic and kinetic data were filtered using a fourth-order low-pass Butterworth filter, with cutoff frequencies set at 15 Hz for kinematic data and 55 Hz for ground reaction force data, based on previous landing biomechanics studies and residual analysis. Marker trajectories collected by the Vicon system were calibrated and truncated; missing trajectories (<50 frames) were interpolated using a cubic spline algorithm in Visual3D, while gaps exceeding 50 frames resulted in trial exclusion. The processed data were exported in C3D format and imported into Visual3D (C-Motion Germantown, MD, United States). A personalized static skeletal model was constructed for each participant. Joint kinematics, including joint angles and center-of-mass trajectory, were calculated using dynamic mapping between markers and anatomical segments. GRF data collected by the Kistler force plate were synchronized with the kinematic data. Inverse dynamics was performed in Visual3D to calculate joint moments and joint reaction forces. GRF and joint reaction force variables were normalized to body weight, whereas joint moments were normalized to body mass. To minimize the influence of movement-duration differences among trials and participants, all time-series data were normalized to 100% of the complete movement cycle. For each participant, the mean value of three successful trials was used for subsequent analysis. Descriptive statistics were used to summarize kinematic and kinetic variables. Data are presented as mean ± standard deviation unless otherwise stated. Given the exploratory and descriptive nature of this sport-specific biomechanical study, statistical comparisons between phases were not performed. Descriptive statistics were used to summarize kinematic and kinetic variables. Data are presented as mean ± standard deviation. The reported differences are descriptive and should be interpreted as phase-dependent trends rather than statistically inferred differences.

### 2.6 Finite element model construction

#### 2.6.1 Geometric reconstruction

One representative participant(age:19 years; height:177.0 cm; body mass:67 kg), whose anthropometric data were comparable to the corresponding mean values of the overall sample,was selected for subject-specific FE model reconstruction. The supporting-side lower limb was scanned in a supine neutral position using spiral CT and MRI with a slice thickness of 0.625 mm.

The DICOM images were imported into Mimics (version 21.0; Materialise NV, Leuven, Belgium). Bone and soft tissues were segmented using thresholding, region growing, and manual mask editing. Preliminary three-dimensional models were generated and exported in STL format. The STL file was then imported into Geomagic Wrap (version 2021; 3D Systems, Inc., Rock Hill, SC, USA), where the Mesh Doctor function was used to repair defects such as non-manifold edges and holes. After mesh simplification and surface fitting, a STEP-format model was exported.

Based on MRI and anatomical data, the cartilage, menisci, and ligamentous structures were constructed in SolidWorks (version 2024; Dassault Systèmes, Vélizy-Villacoublay, France), and the complete assembly was performed. Finally, the CAD model of the knee joint was meshed using HyperMesh (version 2025; Altair Engineering, Inc., Troy, MI, USA). The establishment of the FE model of the knee joint is shown in Figure 3.

**FIGURE 3:**
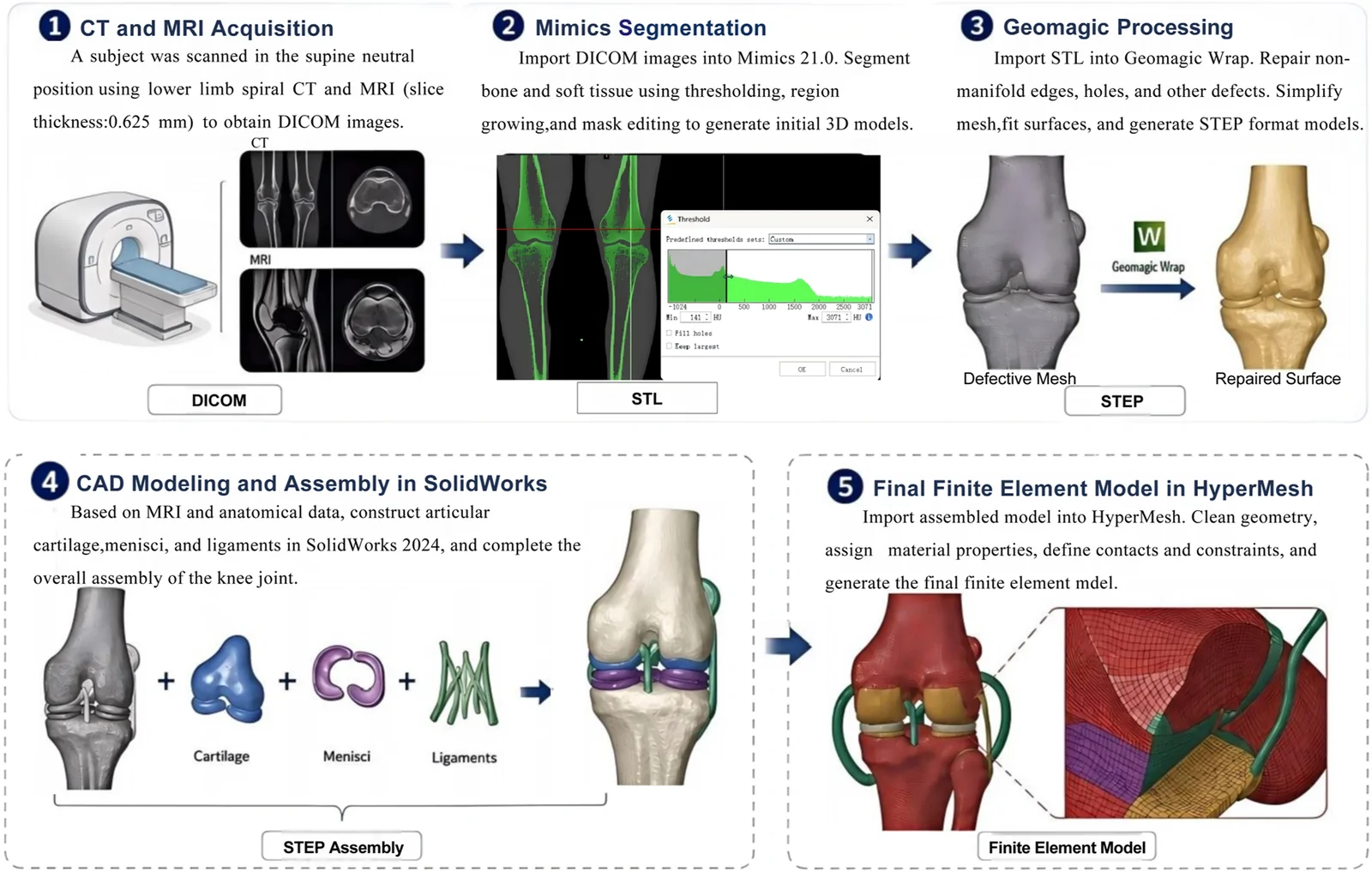
Process of knee joint model construction.

#### 2.6.2 Mesh generation and material properties

The assembled CAD model was imported into HyperMesh for mesh generation. Bone structures were meshed using tetrahedral elements with an approximate element size of 2 mm. Cartilage, menisci, and ligaments were meshed primarily using hexahedral elements, supplemented by tetrahedral elements in geometrically complex regions, with an element size of 1.0 mm. The mesh size selection was based on convergence tests reported in the source literature from which the material properties were adopted, ensuring a balance between computational efficiency and solution accuracy. All tissues were assumed to be homogeneous, isotropic, and linearly elastic, consistent with previous FE studies of the knee joint under short-term loading. Material properties were assigned based on published studies (Shriram et al., 2017; Zhang et al., 2019; Xu et al., 2022; Dong et al., 2014; Bao et al., 2013; Jia et al., 2026). The specific material parameters are shown in Table 1.

**TABLE 1:** Material parameters and element properties of the FE model.

Tissue	Elastic modulus (MPa)	Poisson’s ratio	Element type
Bone	11,000	0.3	Tetrahedral
Cartilage	15	0.475	Hexahedral-dominated
Meniscus	120	0.45	Hexahedral-dominated
MCL	332.2	0.45	Hexahedral-dominated
PL/LCL/ACL/PCL/ALL	345	0.45	Hexahedral-dominated

#### 2.6.3 Boundary and loading conditions

The experimental biomechanical analysis was conducted across the complete movement cycle. Because the P4 showed the greatest knee joint reaction force and represented the most critical period for knee injury risk, FE analysis was performed at the peak loading moment of this phase. To simulate the internal mechanical environment of the knee joint during P4, the distal surfaces of the tibia and fibula were fully constrained, while the femur was allowed to respond to the applied loads. Hard contact was defined between the menisci and cartilage, with a friction coefficient of 0.002. The cartilage-bone interfaces were defined as tie contact, as shown in Figure 4. The loading conditions applied to the FE model were derived from group-average knee joint reaction force data during P4, obtained from inverse dynamics in Visual3D. The force was decomposed into X-, Y-, and Z-direction components and applied to a coupling reference point at the proximal femur. The specific force components were as follows: Fx = 135.56 N, Fy = −1062.43 N, and Fz = −3347.45 N. This loading strategy was used to reproduce the combined compression, shear, and mediolateral loading conditions experienced by the knee joint at the peak landing impact moment. No pretension or pre-strain was applied to the ligaments or other soft tissues in the model, as this study focused on the relative stress distribution among knee structures under peak loading conditions; moreover, the considerable inter-individual variability in ligament pretension and the lack of standardized values in the literature make its reliable implementation challenging.

**FIGURE 4:**
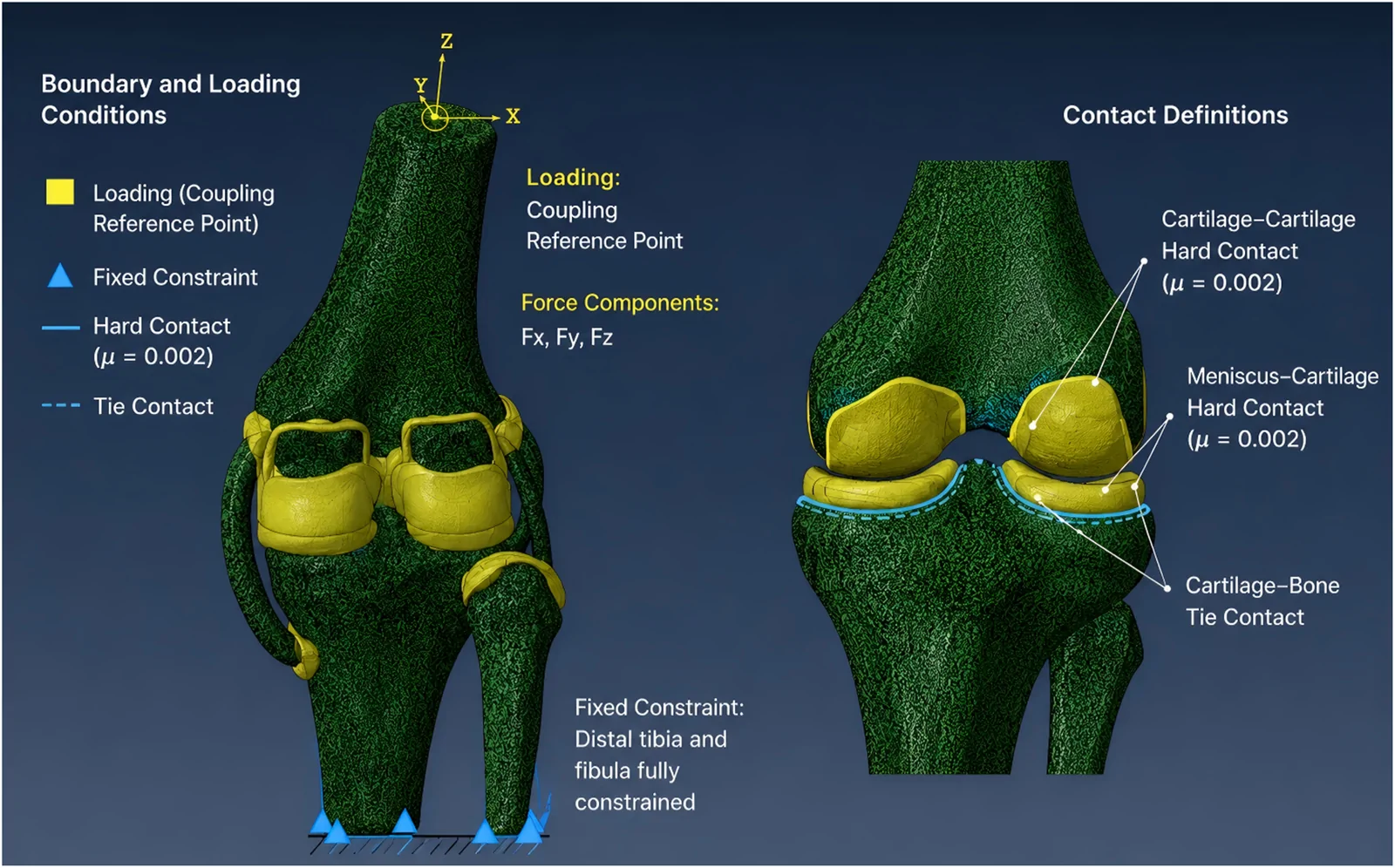
Boundary, loading conditions and contact definitions used in FE analysis.

It should be noted that knee joint moments and individual muscle forces were not directly incorporated as loading inputs in the current FE model, as the primary objective was to evaluate the mechanical stress response of passive knee structures under the predominant joint reaction forces. FE analysis was performed in Abaqus (version 2024; Dassault Systèmes, Vélizy-Villacoublay, France).

#### 2.6.4 FE model validation

To validate the biomechanical reliability of the FE model, a 1,000 N axial compression load was applied to the knee joint, and the resulting tibiofemoral contact area and contact pressure were compared with previously published experimental and computational data (Zhang et al., 2019; Bao et al., 2013; Allaire et al., 2008). The model predicted a total tibial contact area of 1,004.3 mm^2^, a peak tibial cartilage contact pressure of 3.60 MPa, a peak meniscal shear stress of 6.19 MPa, and a tibial cartilage shear stress of 2.46 MPa. These values were within the ranges reported in previous knee FE and cadaveric studies, indicating that the present model had acceptable biomechanical validity for subsequent stress analysis.

## 3 Results

### 3.1 Kinematic

#### 3.1.1 Movement duration

As shown in Figure 5, The durations of the four movement phases showed different durations. P1 lasted 0.369 s, P2 was the shortest phase at 0.278 s, P3 lasted 0.361 s, P4 was the longest phase at 1.639 s. These results indicate that the athletes completed take-off and aerial technical actions within a compressed time window, whereas a longer period was required after landing to absorb impact and restore postural stability.

**FIGURE 5:**
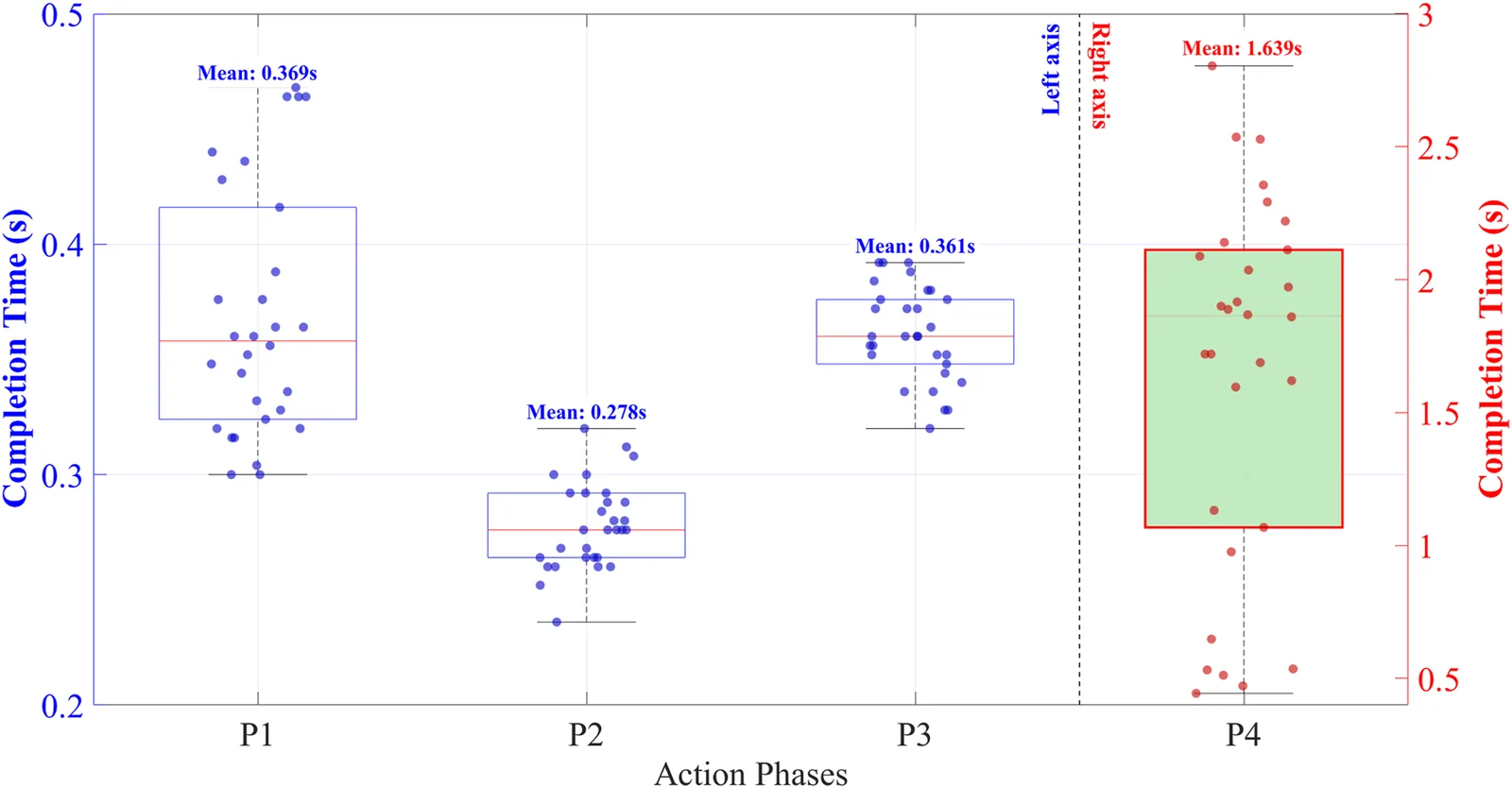
Action completion time.

#### 3.1.2 Center-of-mass height

As shown in Figure 6, During 322C, the center-of-mass height first increased and then decreased. At the beginning of the movement, the center of mass was at its lowest position, 0.49 ± 0.04 m. It then rose rapidly during P1 and P2, reaching a maximum height of 1.67 ± 0.05 m at approximately 28% of the movement cycle. Notably, the center-of-mass height at the hand-foot slap contact moment had not yet reached its peak; instead, it continued to increase after hand-foot slap contact before entering the descent phase. During P4, the center-of-mass height decreased rapidly and then continued to decline slightly, suggesting that the athletes used progressive lower-limb flexion to complete impact absorption and stabilization.

**FIGURE 6:**
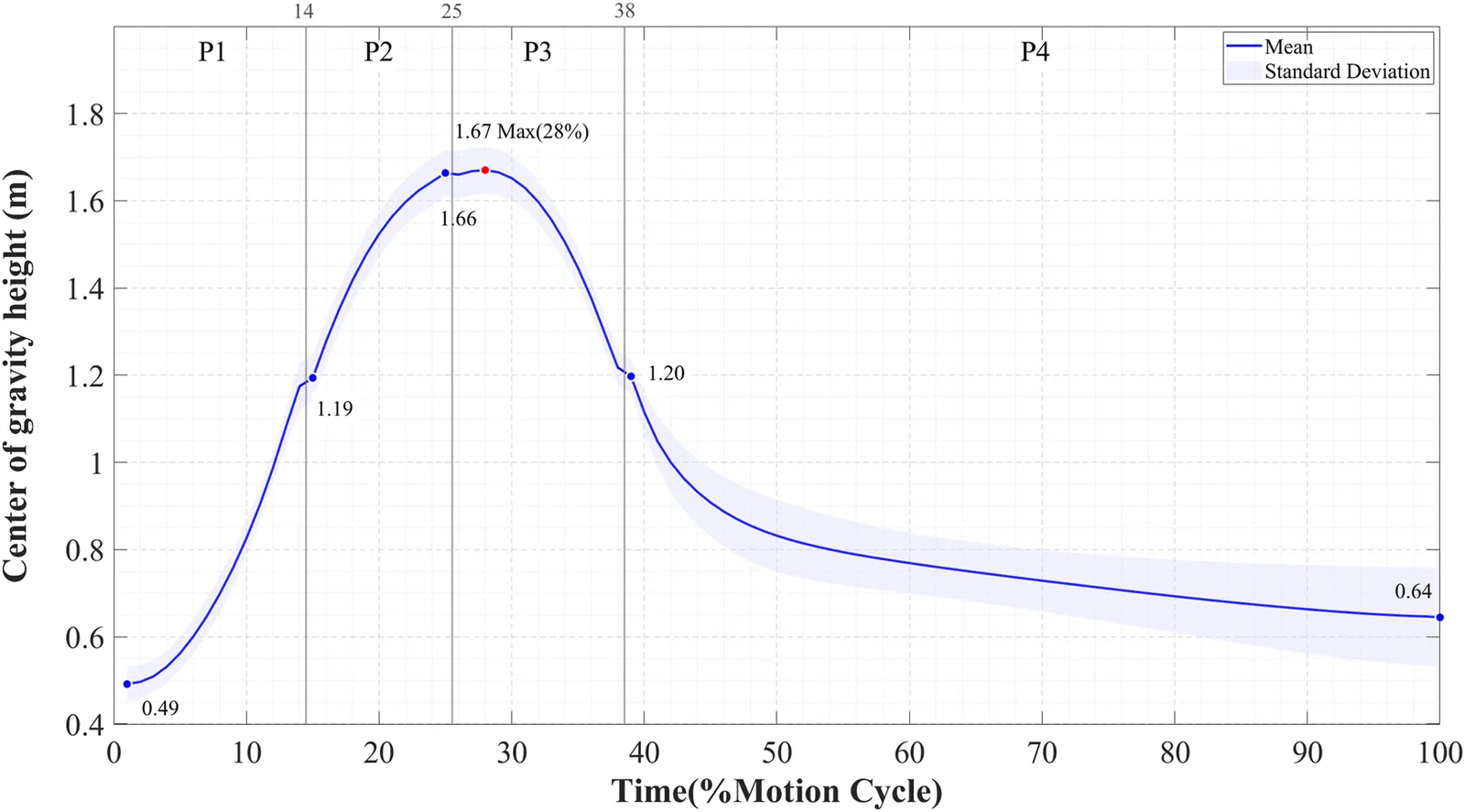
Curve of human center of mass height variation.

#### 3.1.3 Knee angles

As shown in Figure 7, The three-dimensional angular changes of the supporting-side knee joint showed clear phase-dependent characteristics.

**FIGURE 7:**
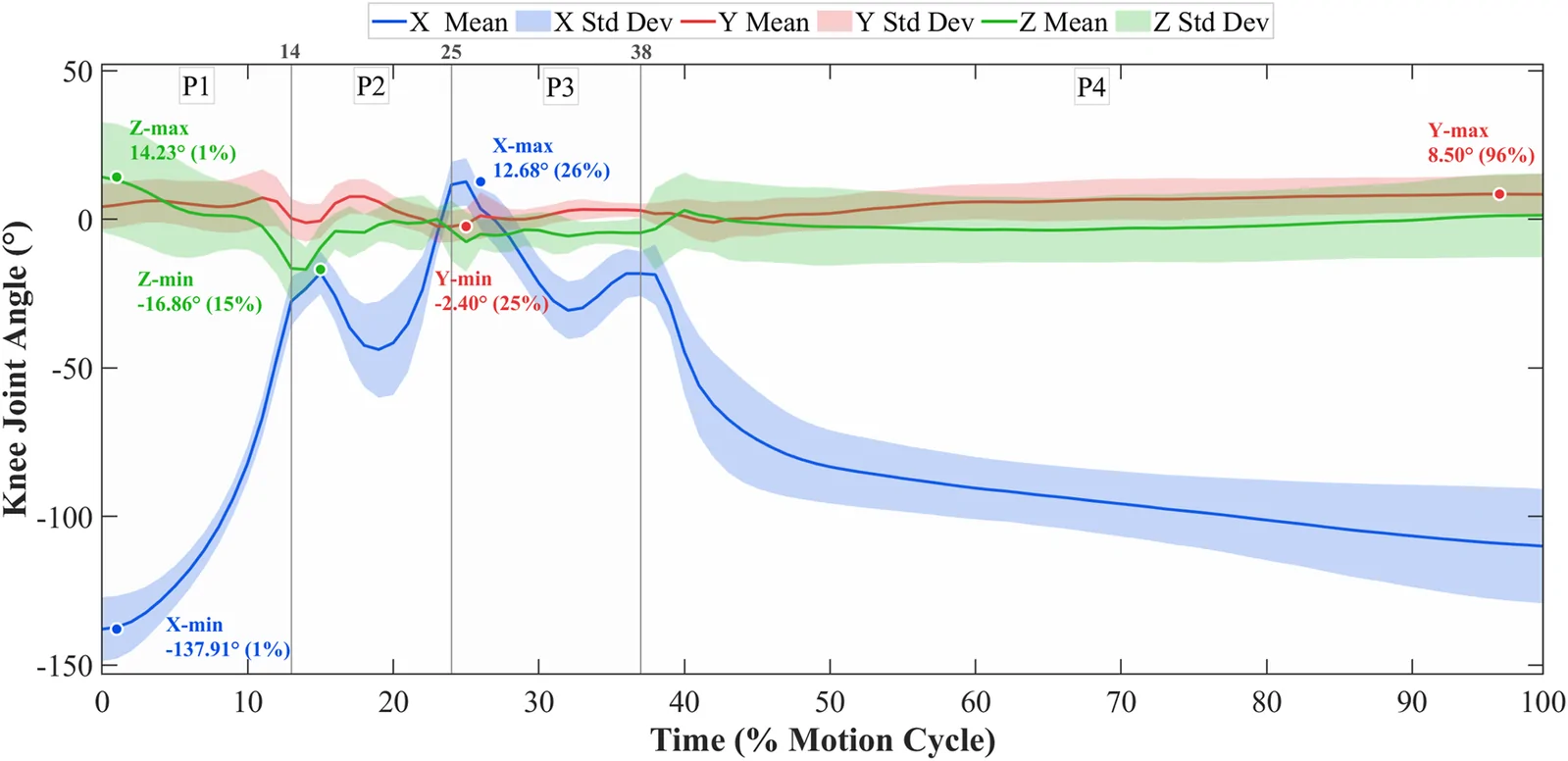
Curve of knee joint angle variation on the supporting side.

During P1, the knee rapidly moved from a deep flexed position toward extension. The initial knee flexion angle was −137.91° ± 10.72°. Frontal-plane adduction-abduction changes were relatively small. In the transverse plane, the knee internal rotation angle decreased from an initial maximum of 14.23° ± 18.51° and gradually changed toward external rotation.

During P2, knee flexion showed a pattern of first decreasing, then rapidly increasing, and finally decreasing again. By the end of this phase, the knee changed from flexion toward extension. Frontal-plane motion remained limited, and the knee adduction angle first increased and then decreased, eventually changing into slight abduction fluctuation. The maximum knee abduction angle was −2.40° ± 5.30° at approximately 25% of the movement cycle. In the transverse plane, the maximum external rotation angle was −16.86° ± 7.48° at approximately 15% of the cycle and then gradually decreased.

During P3, the knee extension angle reached a maximum of 12.68° ± 7.95° at approximately 26% of the movement cycle, then gradually decreased and returned to slight flexion. Frontal- and transverse-plane angles fluctuated within a relatively small range, indicating that the supporting-side knee mainly performed postural adjustments in preparation for landing.

During P4, the knee flexion angle continuously increased, showing a clear flexion-cushioning pattern. In contrast, frontal- and transverse-plane angular changes remained relatively small. The knee adduction angle reached its maximum value of 8.50° ± 6.26° at approximately 96% of the movement cycle. These results suggest that athletes primarily absorbed landing impact through sagittal-plane knee flexion while attempting to maintain frontal- and transverse-plane stability.

### 3.2 Kinetic

#### 3.2.1 Knee moments

As shown in Figure 8, During P1, the supporting-side knee extension moment decreased rapidly and reached its minimum value of 0.41 ± 0.34 N·m·kg^−1^ at 14% of the movement cycle. During same period, the abduction moment changed toward an adduction moment, whereas rotational moments showed relatively small fluctuations. The peak external rotation moment also occurred at approximately 14% of the cycle, with a value of −0.22 ± 0.20 N·m·kg^−1^.

**FIGURE 8:**
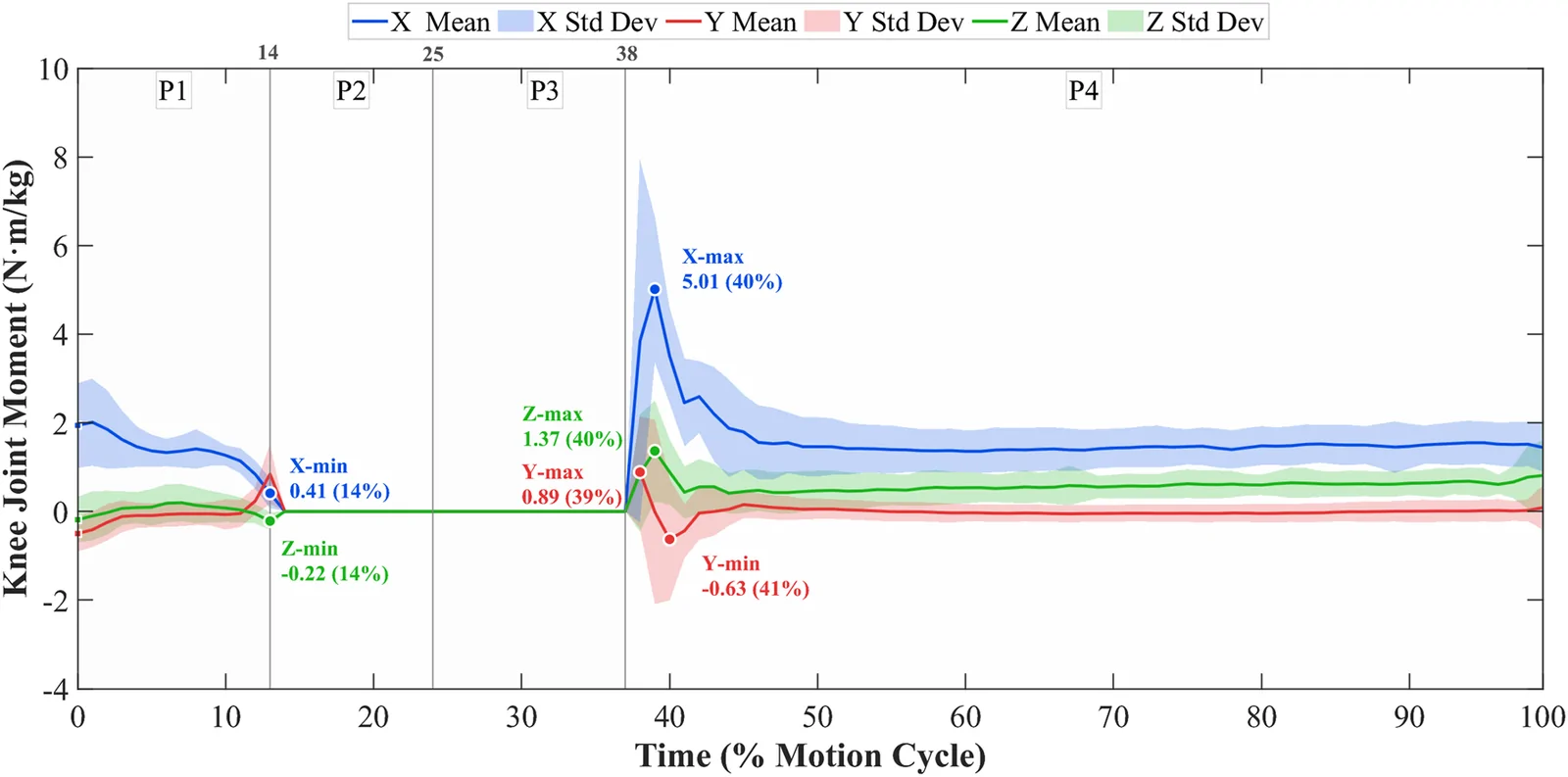
Curve of knee joint moment variation on the supporting side.

During P4, the knee extension moment increased rapidly immediately after ground contact and reached a peak value of 5.01 ± 1.64 N·m·kg^−1^ at approximately 40% of the movement cycle. It then decreased quickly and gradually stabilized. The adduction moment reached a maximum value of 0.89 ± 1.26 N·m·kg^−1^ at approximately 39% of the cycle, then decreased rapidly and changed toward an abduction moment. The abduction moment subsequently increased and then decreased, reaching a maximum magnitude of −0.63 ± 1.37 N·m·kg^−1^ at approximately 41% of the cycle. In addition, the internal rotation moment reached a peak value of 1.37 ± 1.14 N·m·kg^−1^ at approximately 40% of the cycle and then gradually decreased.

Overall, knee joint moments increased substantially in the sagittal, frontal, and transverse planes during landing, indicating that this phase required not only impact absorption but also postural stabilization and rotation braking.

#### 3.2.2 Knee reaction forces

As shown in Figure 9. During P1, the X-direction anterior-posterior joint reaction force showed relatively small fluctuations and remained within approximately 1–2 BW. The Y-direction mediolateral reaction force reached a maximum lateral peak of −9.23 BW and then decreased rapidly. The Z-direction tension-compression reaction force ranged from approximately −7 to 2 BW and mainly showed a compression pattern, increasing rapidly and then gradually decreasing. Toward the end of this phase, the compression load changed into a slight tension load.

**FIGURE 9:**
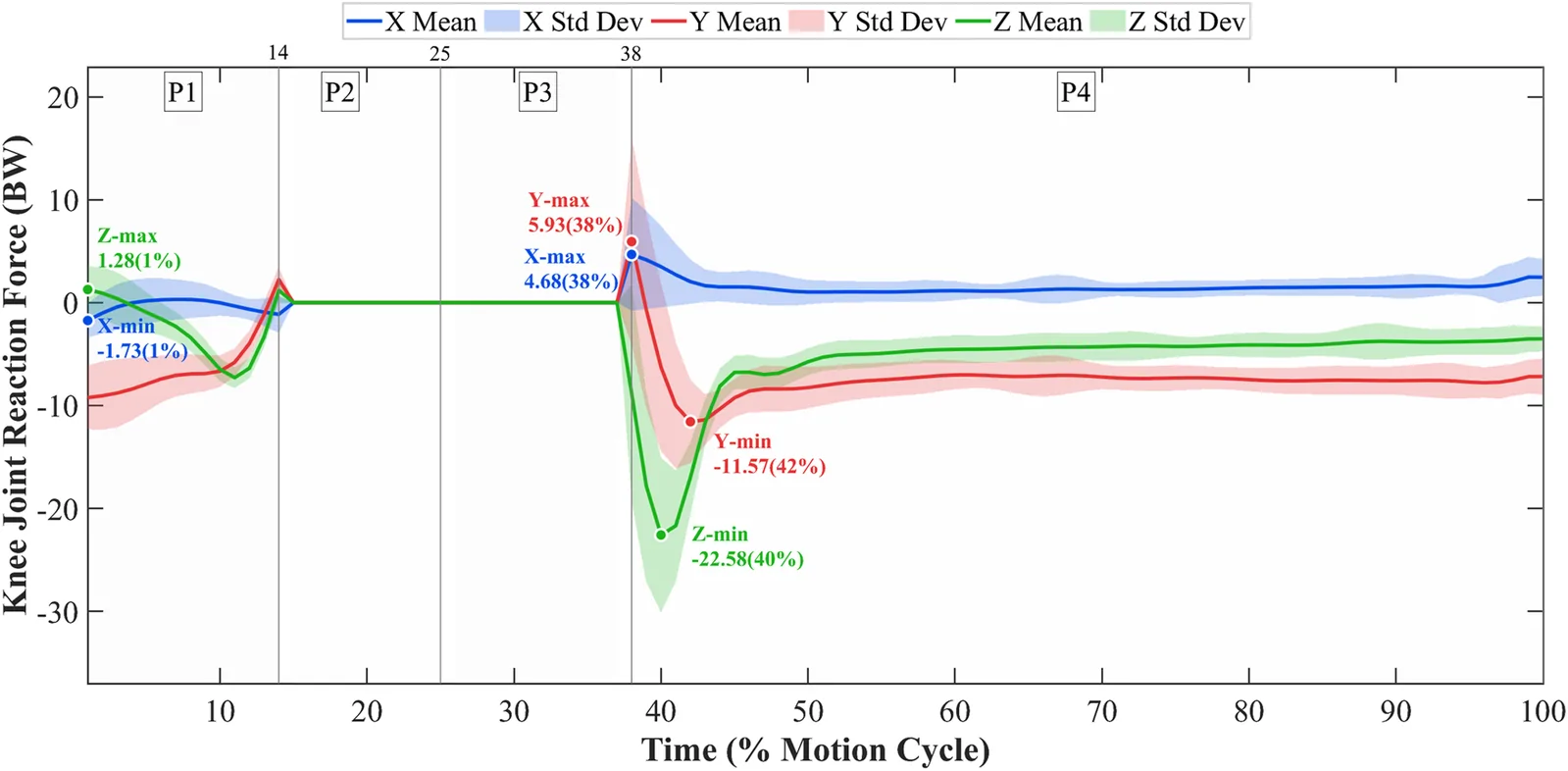
Curve of knee joint reaction force variation on the supporting side.

During P4, knee joint reaction forces increased markedly. The X-direction reaction force reached an anterior peak of 4.68 BW immediately after landing, then decreased rapidly and stabilized within 1–2 BW. The Y-direction reaction force initially showed a large medial force with a peak of 5.93 BW, then quickly changed toward a lateral force, reaching the maximum lateral peak of the entire movement cycle at −11.57 BW around 42% of the cycle. The Z-direction reaction force reached the highest compression load of the entire movement cycle during P4, increasing rapidly after landing and peaking at −22.58 BW around 40% of the cycle, before decreasing quickly and stabilizing at approximately 4 BW.

These results indicate that the P4 involved not only substantial axial compression but also marked anterior shear and mediolateral loading, reflecting a multi-planar coupled loading pattern of the supporting-side knee joint.

#### 3.2.3 Vertical ground reaction force

As shown in Figure 10. At the beginning of the movement, the VGRF was 1.03 ± 0.33 BW. During P1, VGRF fluctuated only slightly and remained relatively stable in the early part of the phase, with a peak value of 1.10 ± 0.24 BW. It then decreased rapidly in the late P1. During P4, VGRF displayed a clear double-peak pattern and reached its main peak at 5.18 ± 1.84 BW before decreasing rapidly and gradually stabilizing.

**FIGURE 10:**
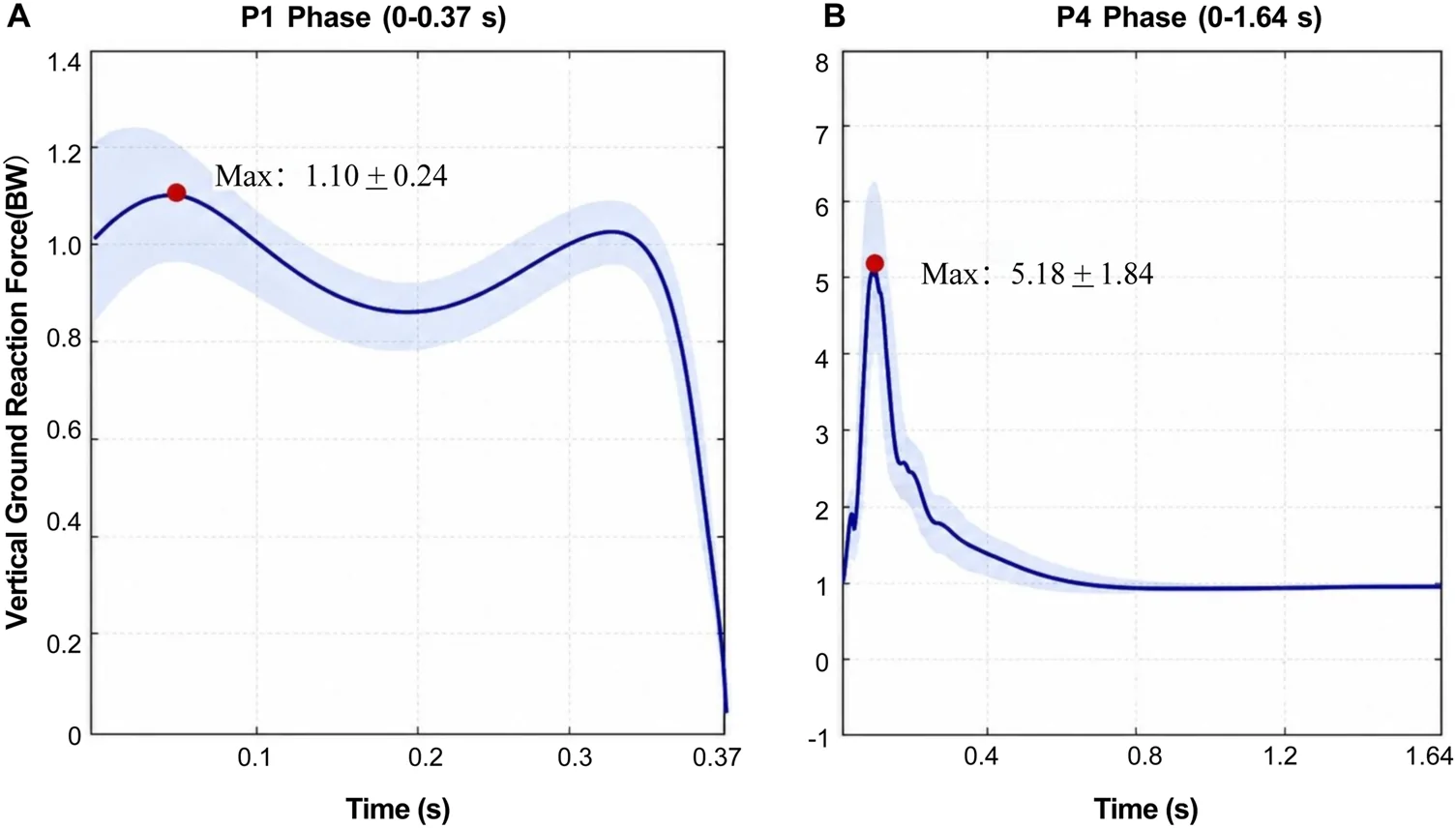
Vertical ground reaction force in **(A)** P1 and **(B)** P4.

The timing of the VGRF peak was highly consistent with the peak compression knee joint reaction force, indicating that the external impact from the ground was an important contributor to the high knee joint loading observed during landing.

### 3.3 Von mises stress distribution

The P4 was the period during which the supporting-side knee joint experienced the most severe mechanical loading. At the peak knee joint reaction force, FE analysis showed region-specific von Mises stress distributions among knee tissues. Overall, ligament and meniscal stress levels were higher than those of articular cartilage. The maximum von Mises stress values were approximately as follows: ACL, 54.24 MPa; PCL, 41.79 MPa; medial meniscus, 23.53 MPa; ALL, 18.89 MPa; MCL, 18.01 MPa; lateral meniscus, 16.46 MPa; PL, 10.66 MPa; LCL, 9.86 MPa; lateral femoral cartilage, 9.62 MPa; and tibial cartilage, 4.30–4.96 MPa.

#### 3.3.1 Femoral cartilage stress

As shown in Figure 11. Von Mises stress in the femoral cartilage was mainly concentrated in the regions contacting the bilateral menisci and patellar cartilage. The highest stress occurred in the lateral femoral cartilage, with a peak value of 9.62 MPa. Stress in the patellofemoral contact region was relatively low. These results suggest that the lateral femoral condyle was the main load-bearing region of the femoral cartilage during landing, whereas the mechanical contribution of the patellofemoral joint was relatively limited at the analyzed instant.

**FIGURE 11:**
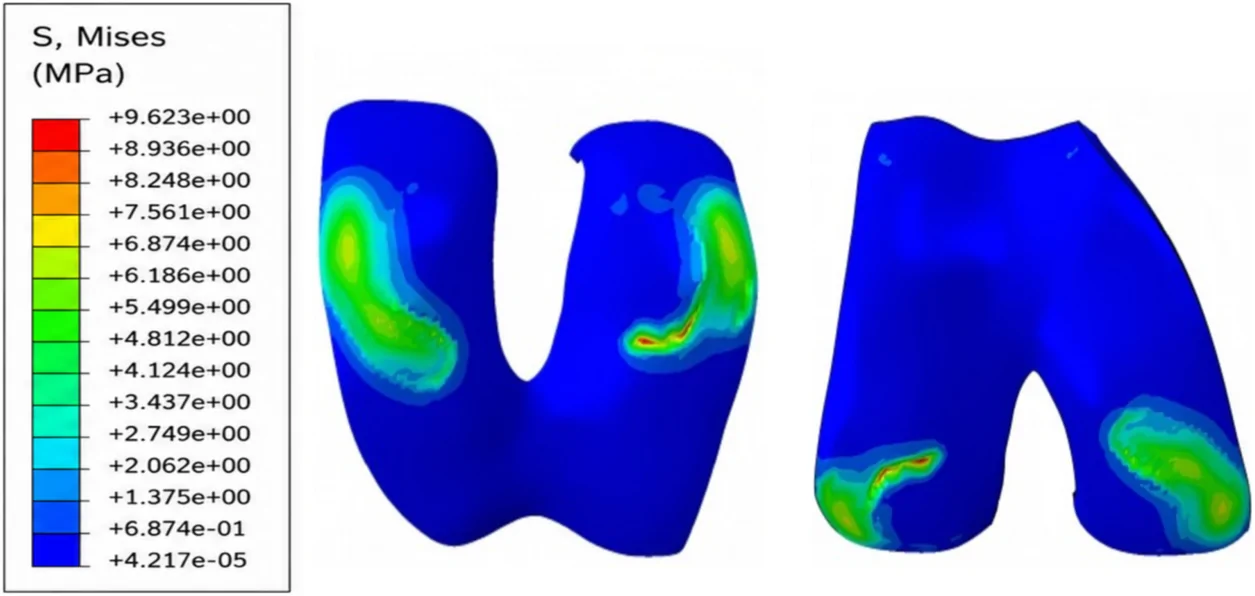
Von Mises stress in the femoral cartilage.

#### 3.3.2 Tibial cartilage stress

As shown in Figure 12. The medial and lateral tibial cartilage showed different stress distribution patterns. Stress in the medial tibial cartilage was mainly concentrated in the anterior region, with a maximum value of 4.96 MPa. Stress in the lateral tibial cartilage was mainly concentrated in the lateral-middle region, with a maximum value of 4.30 MPa. The difference between the two compartments was relatively small, indicating that the tibial cartilage had a certain capacity for load dispersion. However, the anterior region of the medial tibial cartilage still exhibited relatively higher local stress.

**FIGURE 12:**
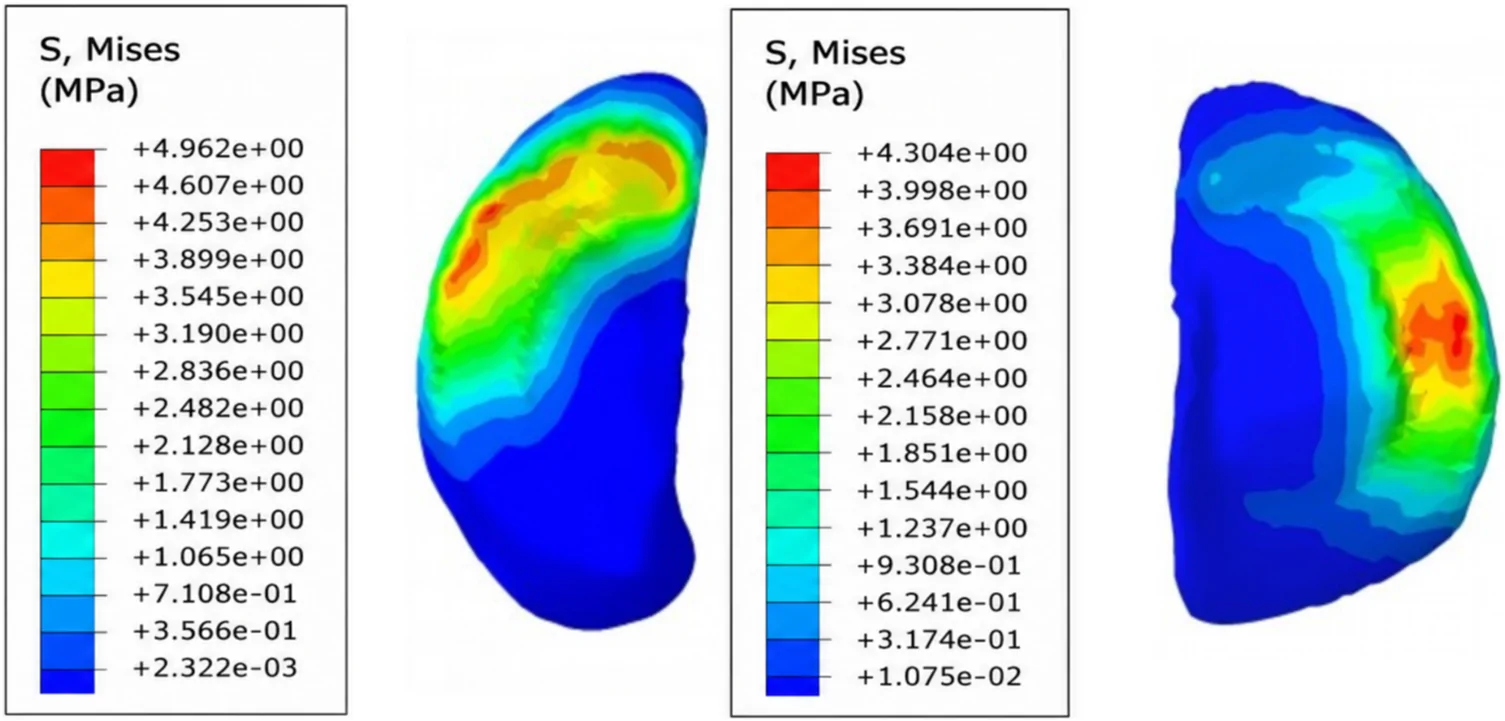
Von Mises stress in the bilateral tibial cartilage.

#### 3.3.3 Meniscal stress

As shown in Figure 13. Both the medial and lateral menisci showed stress concentration mainly in the anterior horn region. In the medial meniscus, stress was most pronounced in the inner part of the anterior horn, with a maximum von Mises stress of 23.53 MPa, and gradually decreased from the anterior horn toward the posterior horn. In the lateral meniscus, stress was also mainly concentrated in the inner anterior horn region, with a maximum value of 16.46 MPa. Overall, meniscal stress showed a pattern of higher stress in the anterior region than the posterior region and higher stress in the inner region than the outer region. These results indicate that the anterior horns of the menisci were important load-bearing regions during P4, particularly under combined compression and rotational shear.

**FIGURE 13:**
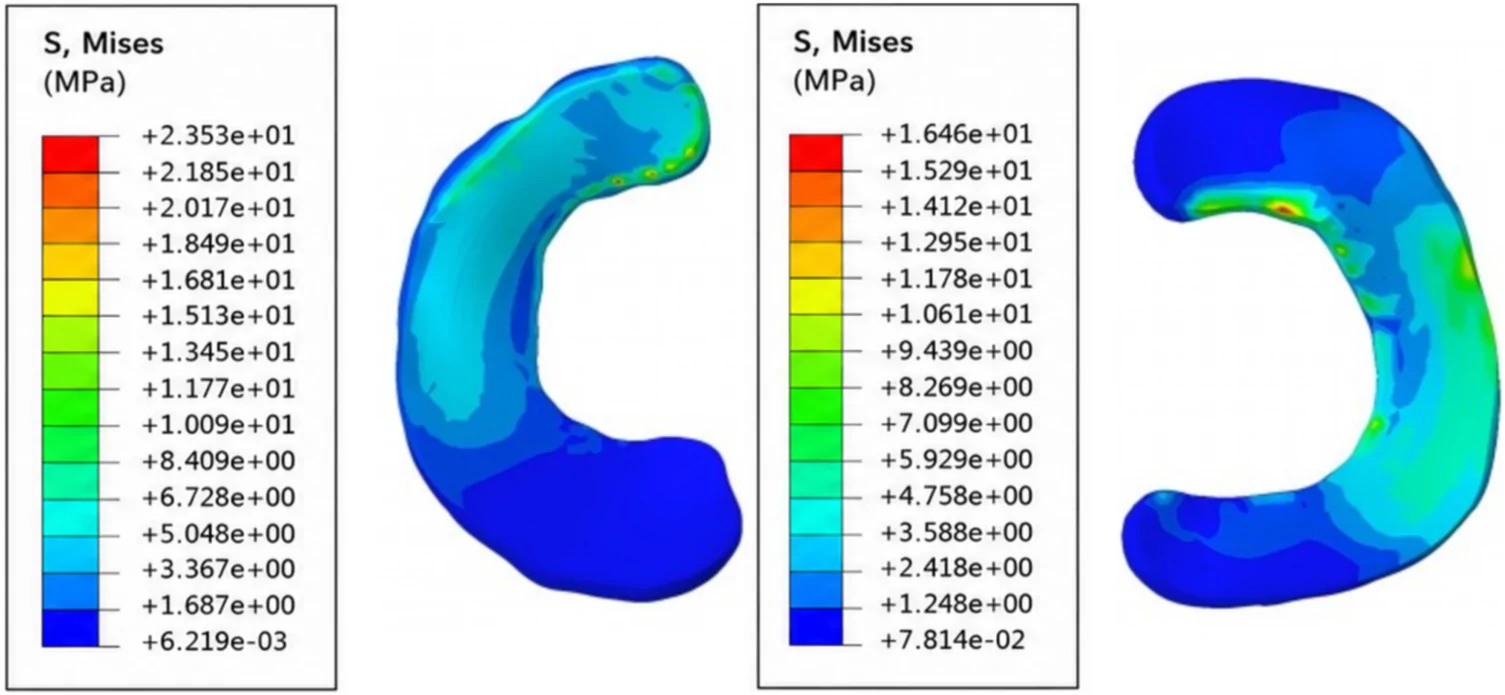
Von Mises stress in the bilateral menisci.

#### 3.3.4 Ligament stress

As shown in Figure 14. Von Mises stress distributions in the knee ligaments were closely related to their anatomical attachments and stabilizing functions.

**FIGURE 14:**
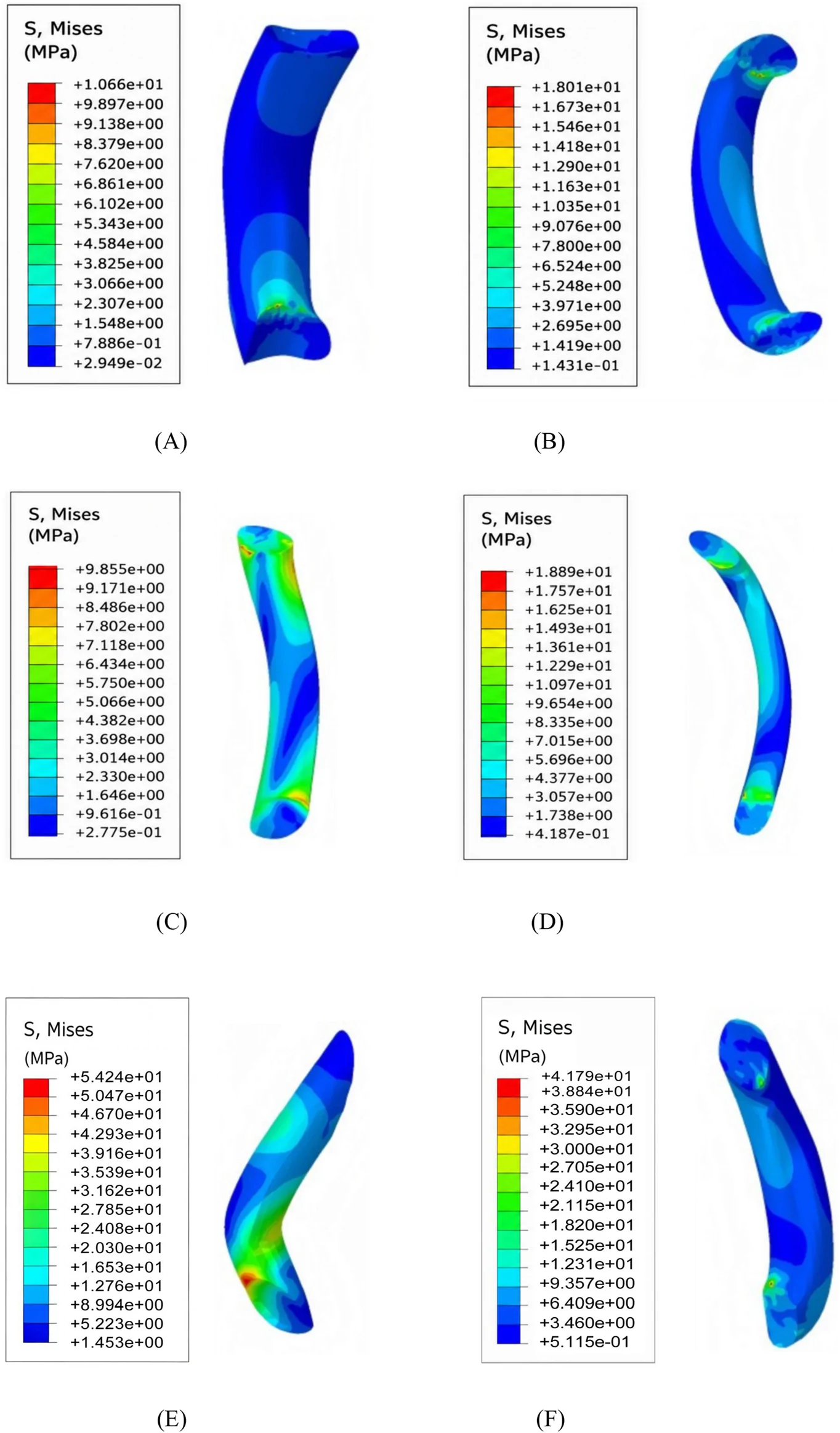
Von Mises stress of the PL, MCL, LCL, ALL ACL and PCL. **(A)** Patellar ligament **(B)** Medial collateral ligament **(C)** Lateral collateral ligament **(D)** Anterolateral ligament **(E)** Anterior cruciate ligament **(F)** Posterior cruciate ligament.

Stress in the PL was mainly concentrated in its lower portion near the tibial tuberosity, with a peak value of 10.66 MPa. MCL stress was mainly concentrated at the bone attachment regions, with the maximum value occurring at the femoral attachment, reaching 18.01 MPa. LCL stress showed a similar attachment-region concentration pattern but a lower overall magnitude, with a peak value of 9.86 MPa. The ALL also showed stress concentration at both attachment regions, with the maximum value at the fibular attachment, reaching 18.89 MPa, suggesting its potential role in resisting rotational instability.

The ACL exhibited the highest stress among all analyzed tissues. Stress was mainly concentrated near the tibial attachment, and the maximum von Mises stress reached 54.24 MPa. PCL stress was also concentrated near the tibial attachment but was lower than that of the ACL, with a maximum value of 41.79 MPa. These results indicate that during landing, the cruciate ligaments, particularly the ACL, were key load-bearing structures for limiting anterior tibial translation and maintaining knee joint stability.

## 4 Discussion

This study integrated kinematic analysis, kinetic analysis, and FE modeling to investigate the load characteristics of the supporting-side knee joint during 322C in competitive Tai Chi.

The experimental analysis covered the complete movement cycle, encompassing the four defined phases, whereas the FE analysis focused on the peak loading moment during P4. The main findings were as follows. First, the movement showed clear phase-dependent biomechanical characteristics: the P1 and P2 were completed within a short duration, whereas the P4 lasted the longest. Second, the supporting-side knee joint showed rapid extension during P1, coordinated flexion-extension adjustment during aerial execution, and continuous flexion during landing. Third, the P4 was associated with markedly increased VGRF, knee extension moment, and compressive joint reaction force, indicating that this phase represented the period of the highest mechanical demand observed among the four phases. Finally, FE analysis revealed non-uniform internal stress distribution, with high stress concentrated in the ACL, PCL, meniscal anterior horns, and lateral femoral cartilage. These findings suggest that the movement imposes distinct phase-dependent loading demands on the supporting-side knee joint, with landing-cushioning being the critical phase for internal tissue loading and potential injury risk.

### 4.1 Kinematic characteristics

The completion time differed across the movement phases of the 322C. The P1 and P2 were relatively short, the P3 was slightly longer, and the P4 lasted the longest. This temporal distribution is generally consistent with previous findings on jumping movements and Tai Chi jump-related techniques (Fang, 2016; Chen, 2017). The P1 represents the initiation and propulsion period of the movement, during which athletes must rapidly generate lower-limb mechanical power to achieve sufficient vertical velocity and angular momentum to obtain sufficient vertical take-off velocity and initial angular momentum for rotation. The short duration of this phase reflects the technical characteristics of rapid force generation and explosive lower-limb extension. The P2 is the airborne striking phase, during which athletes must complete leg swing and hand-foot clap actions while the body is still ascending. Its short duration reflects the compactness and coordination required by the movement. The P3 is slightly longer, representing a transitional period after the slap-kick action, during which the body continues to move upward under inertia and then begins to prepare for landing. During this phase, athletes need to adjust body posture, particularly the relative position of the lower limbs, to establish an appropriate landing configuration.

The P4 showed the longest duration, which is closely related to fundamental biomechanical principles. During landing, the body is subjected to substantial ground impact, and a relatively longer cushioning time is required to dissipate energy through eccentric muscle contraction and joint flexion. This mechanism reduces impact loading, protects the articular cartilage and ligamentous structures, and helps maintain whole-body stability. Previous studies have reported that the peak VGRF during vertical jump landing is generally higher than that during take-off, indicating that greater impact loading requires a longer cushioning period for effective attenuation. Therefore, the temporal pattern observed in this study reflects the kinematic feature of “rapid take-off and slow landing,” which may be regarded as a biomechanical adaptation that allows athletes to balance movement standardization and landing safety when performing high-difficulty skills (Rahikainen, 2015; Karamanoukian et al., 2022).

Furthermore, the “rapid take-off and slow landing” pattern not only reflects rhythm control but also demonstrates the specific lower-limb control requirements imposed by the task. Compared with general jumping movements, the 322C requires athletes to complete take-off, kicking, hand-foot slap, aerial rotation, and stable landing within a short period. At landing, the athlete must rapidly terminate rotational inertia and restore postural stability. Thus, the landing phase bears more complex technical demands and greater mechanical requirements than ordinary jump-landing tasks. These findings suggest that athletes emphasize rapid explosive force production during take-off, whereas during landing they must prolong the cushioning period to absorb impact and reconstruct postural stability. This may explain why the P4 becomes the most critical technical component in the entire movement cycle.

The center of mass of (COM) the human body is defined as the resultant point of the gravitational forces acting on all body segments and serves as a core element in maintaining postural balance and movement coordination. It represents not only the concentration point of body weight but also the convergence of mass and mechanical energy, typically located near the dantian or lumbar region. In Tai Chi, the control and manipulation of the COM are emphasized to achieve both postural stability and mobility, thereby enabling the integration of offensive and defensive functions.

During the execution of 322C, the vertical trajectory of the COM exhibited a typical unimodal curve. During P1, the body reached its maximum squatting position, with the COM descending to its lowest level. Subsequently, the COM rose rapidly as a result of explosive lower-limb extension. Upon entering the flight phase, influenced by the “secondary ascent” effect—whereby inter-segmental momentum exchange and the swing leg action following the slap movement provided additional upward displacement—the COM continued to ascend after the moment of foot-hand contact. This secondary rise elevated the COM further, and it reached its peak at the end of P3.

During 322C, the supporting-side knee joint functions as a key hub in the lower-limb kinetic chain. It plays an essential role in force generation during take-off, posture control during flight, and impact attenuation during landing. Its kinematic behavior directly influences both movement quality and potential injury risk.

During P1, the supporting-side knee joint first increased its flexion angle to complete the countermovement and store elastic potential energy in the lower-limb extensor muscles, thereby contributing to the generation of vertical velocity of the body center of mass. This finding is consistent with previous studies on vertical jumping, which reported a positive association between knee flexion angle during countermovement and jump height (Wang et al., 2024). After the deepest countermovement position, the supporting-side knee joint rapidly shifted from flexion to extension, allowing effective transmission of force through the lower-limb kinetic chain. During this process, the knee joint not only produces the main extension output but also needs to maintain dynamic stability in the frontal and transverse planes to prevent lower-limb alignment deviation and abnormal local loading. Previous research has indicated that excessive lower-limb abduction or internal rotation may increase knee shear force and ligament loading, thereby increasing the risk of sports injury (Ishida et al., 2024). Therefore, in technical training, athletes should reasonably control the lower-limb movement trajectory to achieve a balance between take-off efficiency and knee joint stability.

During P2, the supporting-side knee flexion angle showed a dynamic pattern characterized by an initial decrease, followed by a rapid increase, and then another decrease. The initial reduction in knee flexion was mainly influenced by the inertia generated during take-off propulsion. Subsequently, rapid knee flexion occurred to reaccumulate power and coordinate the swing of the lower limb, thereby ensuring the successful completion of the foot-hand slap action. The final decrease in knee flexion reflected the technical requirement for rapid forward and upward shank movement during the kicking action. This phase requires the knee joint to complete rapid transitions from extension to flexion and then back to extension within a short time, indicating that the knee bears considerable mechanical demand in maintaining movement rhythm and controlling lower-limb inertia. Moreover, because the body is in the airborne state and lacks ground support, the muscles surrounding the knee joint must actively contract to maintain joint stability and reduce additional stress caused by uncontrolled joint oscillation.

During P3, the supporting-side knee flexion angle first increased and then decreased. The initial increase may be attributed to passive backward flexion of the shank under inertia and gravity after completion of the foot-hand slap action. In the later period, the shank actively extended to prepare for ground contact. In this phase, the knee joint is not only responsible for lower-limb posture adjustment but also needs to establish a neuromuscular control pattern for the upcoming landing. Previous studies have shown that athletes often initiate a pre-activation or pre-cushioning strategy during late flight phase, in which the knee enters a slightly flexed position before landing to reduce the effect of neural response delay on landing stability. This strategy may enhance the lower limb’s ability to accommodate impact loading at initial contact. However, insufficient knee flexion may cause the GRF to be concentrated in the knee joint structures, whereas excessive flexion may affect technical standardization and movement quality. Therefore, athletes must seek an optimal balance between the aesthetic and technical requirements of competitive Tai Chi and biomechanical safety.

During P4, the supporting-side knee flexion angle increased rapidly. At this moment, the knee extensor muscles absorbed ground impact mainly through eccentric contraction, thereby reducing the direct transmission of impact loading to articular cartilage, menisci, and ligamentous structures. Previous studies have demonstrated that appropriate knee flexion during landing can effectively reduce ACL tensile stress and lower the risk of landing-related injuries (Su et al., 2009; Ma et al., 2020). In addition, the present study found that angular changes in the frontal and transverse planes of the supporting-side knee joint were relatively small during landing. This indicates that athletes were able to maintain relatively stable knee alignment during the landing process, which may be beneficial for reducing local stress concentration and improving landing stability.

It should be noted that the small angular changes in the frontal and transverse planes do not mean that control in these planes is unimportant. On the contrary, under the condition of single-leg landing combined with rotational inertia braking, small varus-valgus and rotational displacements may reflect active neuromuscular constraints on multi-planar joint motion. This suggests that the control objective of the supporting-side knee joint in this movement is not to eliminate frontal- and transverse-plane motion completely, but rather to limit excessive valgus-varus and rotational deviations under high-impact conditions. Such a strategy helps maintain lower-limb alignment and avoids additional stress on local joint structures.

From a practical perspective, these kinematic characteristics suggest that coaches and athletes should continue to emphasize soft and controlled landing techniques in specific training. Landing patterns such as stiff-knee landing, insufficient knee flexion, or excessively hard ground contact should be avoided. At the same time, frontal- and transverse-plane stability training under single-leg support conditions should be strengthened to improve multi-planar knee control during high-difficulty Tai Chi movements.

### 4.2 Kinetic characteristics

Joint moments reflect muscle force generation and moment regulation during movement and are important indicators of lower-limb power output and cushioning capacity. In the 322C, the supporting-side knee joint contributed to take-off power generation and served as a core structure for impact absorption and stabilization during landing.

During P1, the knee extension moment of the supporting side decreased rapidly. The results of this study showed that after completing the maximum crouch movement, the supporting-side knee joint rapidly transitioned from extension to flexion, generating a large flexion moment that propelled the center of mass upward. Meanwhile, the knee joint reaction forces exhibited distinct phase-dependent characteristics: the anterior-posterior reaction force (X-direction) remained relatively stable within the range of 1–2 BW, indicating limited anterior-posterior shear loading at the knee during P1; the medial-lateral reaction force (Y-direction) reached a peak lateral force (−9.23 BW) at the instant of E5, followed by a rapid decrease, suggesting a certain degree of frontal-plane load regulation during P1; the tension-compression reaction force (Z-direction) was predominantly compression, ranging from −7 to 2 BW, showing a rapid increase followed by a gradual decrease, indicating that the knee joint sustained substantial axial compression loading to facilitate lower-limb power transfer during P1. At the end of this phase, the compression load gradually transitioned into a mild tension, reflecting the rapid reduction in joint loading after the lower limb left the ground.

These changes in knee joint reaction force indicate that during take-off, the supporting-side knee joint not only provides the main power output but also maintains frontal-plane stability and lower-limb alignment to ensure effective force transmission from the proximal to distal segments. If excessive medial-lateral deviation or rotational loading occurs during this phase, the mechanical stress on the menisci and ligamentous structures may increase, thereby elevating the risk of sports injury.

During P4, the kinetic load of the supporting-side knee joint increased markedly, showing a typical high-impact cushioning pattern. At initial contact, the knee joint moment increased rapidly and was highly coupled with the peak VGRF. Previous studies have suggested that eccentric contraction of the knee extensor muscles during landing is an important mechanism for absorbing impact energy and reducing joint injury risk (Pathak and Ahn, 2021; An et al., 2015). In the present study, the VGRF during landing showed a typical double-peak pattern, which is generally consistent with the mechanical characteristics observed in running and jumping movements (Lee and David, 2011). The peak VGRF reached 5.18 BW, which is higher than many ordinary double-leg landing tasks (Hokimoto et al., 2024). This difference is likely attributable to the movement pattern of bilateral take-off followed by single-leg landing, which concentrates the impact load on the supporting side.

The 322C also involves residual rotational inertia at landing. The supporting side must therefore absorb vertical impact while braking rotation and restoring postural stability. Thus, the knee joint does not simply function as a load-bearing joint; it simultaneously participates in impact absorption, postural regulation, and rotational control. This combined mechanical demand explains the high knee joint loading observed during landing.

The joint reaction force components further demonstrated that the landing phase involved multi-planar coupled loading. The anterior-posterior force peaked immediately after landing, suggesting large early anterior shear loading. The mediolateral force changed rapidly from medial to lateral, indicating continuous frontal-plane adjustment for single-leg stabilization. The compression force reached its maximum during landing, suggesting that the femur, tibia, cartilage, and menisci jointly participated in absorbing and transmitting axial impact.

Insufficient lower limb shock absorption capacity or inadequate knee flexion attenuation during landing may induce localized stress concentrations, thereby increasing the risk of injury to the ACL, menisci, and patellofemoral joint. It is important to emphasize that, although the kinematic results revealed relatively small angular changes in the frontal and transverse planes during P4, the kinetic results demonstrated substantial peak variations in the anterior-posterior and mediolateral directions. This indicates that knee joint loading during landing is not limited to vertical impact, but rather constitutes a multi-planar coupled load involving compression, shear, and mediolateral stabilization demands. Consequently, small kinematic angular displacements do not necessarily imply low internal loading; conversely, such restricted motion may reflect active neuromuscular control aimed at limiting hazardous joint displacement and maintaining lower limb alignment stability. Under such conditions combining high impact loading with multi-planar stabilization requirements, key internal structures of the knee joint are more susceptible to localized high tissue loading.

Furthermore, the present study found that the timing of the peak knee joint reaction force was highly consistent with that of the peak VGRF, indicating that ground impact loading is a major contributor to increased internal knee joint loading. Notably, under the single-leg landing mode, the supporting side knee is required not only to absorb impact but also to maintain body balance and postural stability. Thus, its mechanical loading is characterized by high impact and high stabilization demands.

In summary, during the execution of the competitive Tai Chi “322C” maneuver, the supporting side knee exhibits distinct dynamic loading characteristics across different phases: the P1 is dominated by knee extensor moment output and axial compression load transmission, whereas the landing phase is characterized by high-intensity impact absorption and frontal-plane stability control. Notably, the high VGRF and high knee compression reaction force observed during P4 are key factors contributing to the significantly increased knee joint loading. These findings provide critical kinetic evidence for subsequent FE analyses of knee joint stress distribution, cartilage contact pressure, and potential injury risk assessment.

### 4.3 Stress characteristics of the knee joint

Landing, jumping, and rotation are core components of many high-difficulty sports techniques. In competitive Tai Chi, the 322C places high demands on aerial posture control, landing cushioning, and postural stabilization. During landing, the lower-limb joints absorb and dissipate impact energy through coordinated flexion, and the knee joint plays a central role in load transmission and posture regulation (Hewett et al., 2005; Xu et al., 2024). In this phase, the GRF is rapidly transmitted upward through the plantar surface, creating a complex and dynamically changing mechanical environment within the knee joint. Articular cartilage, menisci, and ligaments jointly bear and transmit loads to maintain knee stability and function. Accurate analysis of the mechanical response of the knee joint during dynamic landing can help reveal the biomechanical characteristics underlying movement execution and provide a theoretical basis for technique optimization and training regulation.

FE analysis provides a useful method for quantifying and visualizing internal tissue stress under dynamic loading conditions (Xiang et al., 2023; Makinejad et al., 2013). In the present study, the FE results showed clear anatomical specificity in stress distribution. External impact and joint reaction forces were not uniformly transmitted within the knee; instead, stress was concentrated in structures responsible for stabilization and load distribution. This finding is consistent with the kinetic results showing high impact and multi-planar stabilization demands.

Von Mises equivalent stress serves as a comprehensive evaluation indicator that reflects the overall mechanical response of materials under complex stress states, effectively capturing the combined effects of stress components from various directions within the tissue. Under the dynamic loading conditions employed in this study, Von Mises equivalent stress enables a more holistic assessment of the overall mechanical load levels experienced by various tissues within the knee joint under complex stress states, thereby providing an objective basis for determining their mechanical response characteristics and structural adaptive capacity.

Under the dynamic loading condition, Von Mises stress in the femoral cartilage was predominantly concentrated in the lateral condyle region, with a maximum value of 9.62 MPa, while the stress level at the patellofemoral joint interface was relatively lower. This characteristic directly corroborates the mechanical pattern of lateral compartment dominance in load bearing during P4 of the maneuver. As the movement necessitates counteracting rotational inertia, the lower limb mechanical axis shifts laterally, rendering the lateral femoral condyle the primary load-bearing site subjected to both vertical impact and rotational shear, whereas the mechanical involvement of the patellofemoral joint remains relatively limited during this phase. Compared with the peak Von Mises stress in femoral cartilage reported during drop landing maneuvers in the general population (Xiang et al., 2023),the stress value measured in this study was approximately twice as high, suggesting a high mechanical loading imposed on the femoral cartilage by this highly demanding turning maneuver. This discrepancy primarily originates from the complex loading characteristics inherent in the 322C maneuver, in which the knee joint is required not only to withstand vertical impact but also to overcome substantial rotational torque generated by rapid body rotation, thereby subjecting the articular cartilage to a considerably more intricate mechanical environment.

Medial tibial cartilage stress was slightly higher than lateral, suggesting effective load sharing through material properties and bony support to balance overall joint loading (Li et al., 2001; Devaraj et al., 2020). Nevertheless, the local stress concentration in the anterior medial tibial cartilage suggests that repeated exposure to this landing pattern may contribute to may contribute to cumulative cartilage loading, particularly under fatigue or poor technical execution.

Overall, the stress distributions in the femoral and tibial cartilages during P4 were not entirely consistent, characterized by localized stress concentration on the lateral femoral condyle and a relatively dispersed stress pattern on the tibial side. This discrepancy may be attributed to differences in articular contact geometry, meniscal involvement, and the load-sharing mechanism between cartilage and surrounding soft tissues. Specifically, the rotational braking action combined with single-leg support imposes elevated contact pressure on the lateral femoral condyle during postural adjustment, whereas the tibial platform exhibits a pronounced load-diffusing capacity mediated by the meniscus and articular cartilage. Nonetheless, the localized high-stress regions identified at the lateral femoral condyle and the anterior medial tibial cartilage suggest that these sites may be predisposed to chronic cartilage degeneration, particularly under conditions of suboptimal movement execution or repetitive training in a fatigued state.

In both menisci, von Mises stress was predominantly concentrated in the inner region of the anterior horns. This distribution pattern aligns with previous reports and reinforces the crucial role of the meniscal anterior horns in attenuating rotational loads (Pena et al., 2006; Xu et al., 2023). This specific stress concentration is directly attributable to the mechanical demands of the maneuver. During landing, internal rotation of the knee subjects the anterior horns to simultaneous compression from the femoral condyles and traction from tibial rotation, generating combined shear-tensile stress that localizes to the inner region. Given the poor vascularity and limited healing capacity of the meniscal anterior horns (Dong et al., 2014), chronic exposure to such high stress may elevate the risk of meniscal tearing. This finding is consistent with clinical observations that meniscal injuries in martial arts athletes frequently involve the anterior horn, implicating rotational loading from turning maneuvers as a key pathogenic factor. Notably, the higher stress observed in the medial anterior horn compared to its lateral counterpart suggests that the medial compartment bears a greater local tissue burden during shock absorption and stability control. Considering the region’s limited reparative capacity, repeated exposure to this high-stress environment may provide a mechanical basis for anterior horn wear or tearing in competitive Tai Chi athletes.

Ligaments, as critical passive stabilizers of the knee joint, exhibit Von Mises stress levels that directly reflect the tensile loads imposed during the restriction of excessive joint displacement. In this study, the stress distribution across ligaments closely matched the stability demands of the movement. The ACL exhibited the highest von Mises stress among all observed structures, reaching a peak value of 54.24 MPa, with stress markedly concentrated at its tibial attachment. This finding is directly attributable to the anterior shear force sustained by the knee during landing. Under the combined effects of vertical impact and rotational torque, the tibia tends to translate anteriorly relative to the femur. As the primary restraint against anterior tibial translation, the ACL consequently bears a substantial tensile load (Hewett et al., 2005; Shi et al., 2025). Furthermore, the high-stress state of the ACL aligns with the clinical observation of frequent cruciate ligament injuries in martial arts athletes, confirming that turning landing maneuvers impose considerable mechanical demand on the ACL (Li, 2021). In contrast, the PCL exhibited relatively lower stress, primarily serving to resist posterior tibial shear and thus bearing a smaller overall mechanical burden.

The MCL showed a maximum Von Mises stress of 18.01 MPa, with stress concentrated mainly at its femoral attachment, whereas the LCL experienced lower stress levels. This medial-lateral disparity reflects the mechanical characteristic of greater medial compartment loading during landing. The associated knee valgus moment imposes higher tensile loads on medial structures, with the MCL acting to counteract valgus force and thereby maintain medial joint stability. Its femoral attachment, as the site of stress concentration, represents a potential regions of elevated mechanical demand. Overall, aside from the ACL, the MCL and other rotation-stabilizing structures also sustain considerable loads during P4, indicating that the internal load distribution of the knee is not dominated by a single overloaded structure. Instead, a complex high-load network is formed, with the ACL as the central hub, complemented by the anterior horn of the meniscus and the tibiofemoral contact regions. This multi-structure load-sharing pattern suggests that, under long-term sport-specific training, the risk of knee injury may manifest as a combination of acute damage to a single tissue and chronic degeneration across multiple tissues.

Integrating the experimental and FE results, we propose the following mechanistic sequence of knee loading during P4 of the 322C: 1) Single-leg impact concentrates external loads, leading to a rapid VGRF rise; 2) Residual rotational inertia and postural reconstruction impose anterior shear and mediolateral demands; 3) The knee primarily uses sagittal flexion to absorb shock while maintaining frontal/transverse stability; 4) Under high-impact, multi-planar demands, internal loads preferentially concentrate in structures responsible for anterior restraint, rotation control, and contact force distribution (i.e., ACL, meniscal horns, focal cartilage regions). Compared with standard vertical jumps, this movement places substantially higher demands on the internal stabilizing structures of the knee, explaining why even externally “well-controlled” landings can still produce significant internal stress concentrations.

It should be acknowledged that the FE model was based on a single representative participant. Although this participant’s anthropometrics were comparable to the group means, the absolute stress values should be interpreted with caution and are not intended to represent the exact tissue loads experienced by all athletes. Rather, the FE analysis provides qualitative insights into possible stress-distribution patterns and identifies potential sites of mechanical vulnerability. Future studies with a larger cohort are needed to verify the generalizability of these findings.

## 5 Limitations

Several limitations should be acknowledged. The sample included only ten male athletes, and the FE model was based on a single participant, so absolute stress values should not be generalized. Soft tissues were modeled as homogeneous, isotropic, and linearly elastic materials, and a formal mesh convergence analysis was not conducted. These simplifications may affect absolute stress magnitudes but do not compromise the main conclusions regarding stress concentration locations.

## 6 Conclusion

Building on previous biomechanical research, this study further characterized the phase-dependent loading pattern of the supporting knee and linked external multiplanar loading characteristics with internal tissue stress distributions. This study integrated motion analysis, inverse dynamics, and subject-specific finite element analysis to investigate the phase-dependent loading characteristics of the supporting-side knee joint during the flying kick with a 180° inward turn (322C) in competitive Tai Chi. The landing-cushioning phase represented the greatest mechanical challenge, characterized by high external impact and non-uniform internal stress concentrations, particularly in the ACL, meniscal anterior horns, and lateral femoral cartilage. These findings improve the understanding of knee loading mechanisms during high-difficulty Tai Chi movements and provide a biomechanical basis for technique optimization, injury prevention, and sport-specific training.

## Data Availability

The raw data supporting the conclusions of this article will be made available by the authors, without undue reservation.
